# A Rare STXBP2 Mutation in Severe COVID-19 and Secondary Cytokine Storm Syndrome

**DOI:** 10.3390/life12020149

**Published:** 2022-01-20

**Authors:** Daniel D. Reiff, Mingce Zhang, Emily A. Smitherman, Melissa L. Mannion, Matthew L. Stoll, Peter Weiser, Randy Q. Cron

**Affiliations:** Department of Pediatrics, Division of Rheumatology, University of Alabama at Birmingham, Birmingham, AL 35233-1711, USA; danielreiff@uabmc.edu (D.D.R.); mizhang@uabmc.edu (M.Z.); Emily.Smitherman@peds.uab.edu (E.A.S.); mmannion@uabmc.edu (M.L.M.); mstoll@uabmc.edu (M.L.S.); pweiser@uabmc.edu (P.W.)

**Keywords:** hemophagocytic lymphohistiocytosis, macrophage activation syndrome, cytokine storm syndrome, COVID-19, genetics, mutation, natural killer cell, cytolysis, degranulation

## Abstract

Background: Primary (familial) hemophagocytic lymphohistiocytosis (pHLH) is a potentially lethal syndrome of infancy, caused by genetic defects in natural killer (NK) cell and CD8 T cell cytotoxicity, leading to hyperinflammation, elevated cytokine levels, and a disorganized immune response resulting in multi-organ system failure and frequently death. Secondary HLH (sHLH) can be triggered in the setting of malignances, diseases of chronic immune system activation, or by infectious etiologies. While pHLH is usually a result of homozygous gene mutations, monoallelic hypomorphic and dominant-negative mutations in pHLH genes have been implicated in sHLH. Coronavirus disease 2019 (COVID-19) has been an omnipresent viral infection since its arrival, and severe cases can present with cytokine storm and have clinical features and laboratory findings consistent with sHLH. Herein, we report an adolescent with severe COVID-19, decreased NK cell function, and features of sHLH. Her genetic evaluation identified a monoallelic missense mutation in the pHLH gene *STXBP2*, and NK cell assays of her blood showed decreased cytolysis and degranulation ex vivo. Methods: Patient data was extracted through an electronic medical record review. Using a lentiviral approach, the patient’s *STXBP2* mutation and wild-type (WT) *STXBP2* were separately transduced into the NK-92 human NK cell line. The WT and mutant *STXBP2* transduced NK-92 cells were stimulated with NK-sensitive K562 erythroleukemia target cells in vitro, and NK cell degranulation and cytolysis were measured via CD107a expression and Live/Dead near-IR dye, respectively. Results: Compared to WT *STXBP2,* the patient’s *STXBP2* mutation caused significantly decreased NK cell cytolysis and associated degranulation in vitro. Conclusion: These findings add weight to the hypothesis that some severe cases of COVID-19 may be accompanied by sHLH and hyperinflammation, especially in the setting of heterozygous pHLH genetic mutations. This has implications both diagnostically and therapeutically for severe COVID-19.

## 1. Introduction

Since its appearance on the world stage in late 2019, severe acute respiratory syndrome coronavirus 2 (SARS-CoV-2) causing coronavirus disease 2019 (COVID-19) has affected a wide swathe of the world population, causing a significant burden of morbidity and mortality in infected individuals. COVID-19 infection severity varies widely from asymptomatic infection, mild-to-moderate symptomology treated in the outpatient setting, to severe end-organ and respiratory failure leading to death or long-term disability. Older age, underlying chronic disease, obesity, and concurrent immunosuppression are associated with increased risk of mortality and severe disease [1]. Within the subset of severe COVID-19, there is increasing evidence for a hyperinflammatory response similar to that seen in cytokine storm syndromes (CSS) and secondary hemophagocytic lymphohistiocytosis (sHLH), characterized by thrombocytopenia, lymphopenia, elevated D-dimer levels, low fibrinogen, elevated lactate dehydrogenase (LDH), elevated liver enzymes, elevated ferritin, and elevation in cytokine/chemokine levels—including IL-1β, IL-6, IL-18, and interferon-gamma (IFNγ) [2]. sHLH is associated with conditions of chronic immune dysregulation, including chronic rheumatic diseases (where it is typically called macrophage activation syndrome (MAS)) and hematologic malignancies. However, certain infectious triggers, including Epstein–Barr virus (EBV) and other members of the herpesvirus family, as well as severe influenza strains are also keen mediators of HLH [3]. It is clear that COVID-19 can serve as a similar infectious trigger of HLH in a certain portion of the infected population [2], but it is unclear as to why certain younger people without known risk factors are severely affected and others are relatively spared. Homozygous genetic mutations, altering natural killer (NK) cells and CD8 T cells, in the perforin-mediated cytolytic pathway (*RAB27A*, *PRF1*, *UNC13D*, *STX11*, *STXBP2*, etc.) can result in the familial/primary form of HLH (pHLH), leading to unregulated immune activation, severe hyperinflammatory disease early in life, and ineffective negative feedback of the immune response [4]. In later onset sHLH, there is growing identification of heterozygous mutations, hypomorphic and partial or complete dominant-negatives, leading to a similar presentation as pHLH, but occurring later in life with a higher threshold for disease [5]. These perforin-mediated cytolytic pathway heterozygous pHLH gene mutations (e.g., *PRF1*, *UNC13D*, *AP3B1*) have been reported in some cases of severe COVID-19 with HLH features [6]. In this report, we present an 18-year-old female with severe COVID-19 and features of HLH, who was found to have a rare (0.036%, Genome Aggregation Database) heterozygous *STXBP2* (c.1286C > T, p.Ala429Val) missense mutation and defective NK cell killing. Her mutation was further explored by lentiviral over-expression in the human NK-92 cell line and comparing its effect on NK cell degranulation and K562 target cell lysis in vitro, relative to over-expression of wild-type (WT) *STXBP2* (control) in NK-92 cells. These findings add to the existing literature of heterozygous pHLH gene mutations contributing to infection-triggered sHLH, improving our understanding of their modulatory effect on the immune response to infectious disease and specifically COVID-19 severity.

## 2. Materials and Methods

### 2.1. Study Approval

This study using de-identified patient data was conducted using the UAB Institutional Review Board approved protocol, number 120907003. Informed consent and a HIPAA release were obtained from the patient and family for participation in this study.

### 2.2. Patient Data

The patient’s clinical course, therapeutics, laboratory values, and post-hospitalization care were obtained via an electronic medical record review using an Institutional Review Board approved protocol. The germline (buccal swab) *STXBP2* mutation was identified via a commercial (Invitae, San Francisco, CA, USA) genetic panel screening for pHLH gene variants. 

### 2.3. DNA Constructs

cDNA encoding WT human *STXBP2* was previously generated by reverse transcription from RNA of the human NK-92 NK cell line [4]. The cDNA was previously cloned into a lentiviral expression vector, and the WT sequence was confirmed by Sanger sequencing. The patient-derived *STXBP2* mutant cDNA (c.1286C > T) was generated from the WT *STXBP2* cDNA by site-directed mutagenesis and confirmed by sequencing [4]. The lentiviral expression vector, z-368-ΔNP, and the packaging plasmid, Δ8.91, were provided by Dr. Philip Zoltick (The Children’s Hospital of Philadelphia) [7]. Both WT and mutant *STXBP2* cDNAs were independently subcloned into z-368-ΔNP to generate recombinant lentiviruses. These viruses were separately transduced into NK-92 cells. As the lentiviral vectors also contained the gene for green fluorescent protein (GFP), transduction efficiencies were monitored by co-expression of GFP as detected by flow cytometry (FCM) (LSR II, BD Biosciences). 

### 2.4. Lentiviral Preparation and Transduction

HEK293T cells were transfected with the respective z-368-ΔNP–expression constructs along with Δ8.91, lentiviral production was concentrated using the Lenti-X concentrator reagent (Clontech, Mountain View, CA, USA), and NK-92 cells were infected with lentiviruses as previously described [8].

### 2.5. Cytotoxicity and Degranulation Assays

The NK-sensitive K562 erythroleukemia target cells were labeled by the cell tracer dye eFluor 450 (eBioscience) 12 h prior to the cytotoxicity assay [9]. The lentiviral-transduced NK-92 cells and labeled K562 target cells were mixed together and stained with Live/Dead near-IR dye (Invitrogen) and analyzed by FCM, as described previously [8]. Anti-human CD56-PE-Cy7 and CD107a-PE monoclonal antibodies (mAbs) were purchased from eBioscience (San Diego, CA, USA). For degranulation assays, the transduced NK-92 cells were mixed with K562 cells, and incubated in the presence of fluorochrome-conjugated anti-CD107a Ab; K562 target cells served as a stimulus for NK-92 cell degranulation [8]. To identify NK-92 effector cells, cells were stained with fluorochrome-conjugated anti-CD56 mAb after incubation with K562 stimulator cells and CD107a cell surface expression on NK-92 cells was detected by FCM as previously described [10]. 

### 2.6. Statistical Analyses

Statistical analyses were performed with GraphPad Prism 6 (GraphPad Software, La Jolla, CA, USA) software. Two-way ANOVA analysis was used to calculate *p* values (α = 0.05) for the NK-92 cell cytotoxicity assays.

## 3. Clinical History

An 18-year-old female with a past medical history of obesity developed a slight cough at home. Over the first three days of illness, her cough continued and she developed epigastric pain, vomiting, and diarrhea. After a syncopal episode on illness day 3, she presented to a local hospital for evaluation and was found to be positive for SARS-CoV-2 on polymerase chain reaction (PCR) nasal swab; of note, she was unvaccinated against SARS-CoV-2 at the time of illness onset. She was also noted to be significantly hypotensive and tachycardic with signs of cardiac dysfunction. Her echocardiogram showed an ejection fraction of 37% and a pericardial effusion; levels of brain natriuretic peptide (BNP) were elevated, and blood gases showed significant acidosis with peak lactate of 16 mmol/L (normal 0.7–2.1). Baseline lab values at initial outside hospital presentation are shown in Table 1. With these findings, the patient was intubated, had a pericardial drain placed, was started on three vasopressor medications for refractory hypotensive shock, and transferred to our tertiary care institution for possible initiation of extra corporeal membrane oxygenation (ECMO). There was no reported family history of severe infections, rheumatologic disease, cardiac failure/dysfunction, or other chronic medical conditions.

On arrival to our institution on day 5 of illness, the patient continued to decline in clinical status and was started on broad-spectrum antibiotics for potential secondary infections, as well as remdesivir 100 mg daily (1 mg/kg) and 8 mg dexamethasone daily (0.08 mg/kg) for management of active SARS-CoV-2 infection. Initial laboratory workup was concerning for significant myocardial dysfunction with troponin I level of 90.1 ng/mL (normal < 0.05), BNP 1498 pg/mL (normal 130–250), and an echocardiogram showing severely diminished left ventricular dysfunction. Inflammatory markers were elevated with an initial C-reactive protein (CRP) of 2.95 mg/dL (normal < 0.50) erythrocyte sedimentation rate (ESR) of 47 mm/h (normal < 20), and ferritin level of 1442.9 ng/mL (normal 5.5–67.4). Due to myocardial injury and significant inflammation, the patient was also started on anakinra (recombinant human interleukin-1 receptor antagonist) 100 mg q6 hours (intravenous, followed by subcutaneous) on day 6 of illness. On day 8 of illness, she was cannulated for ECMO and started on continuous renal replacement therapy (CRRT) in the setting of decreasing urine output, rising creatinine, and volume overload. 

**Figure 1:**
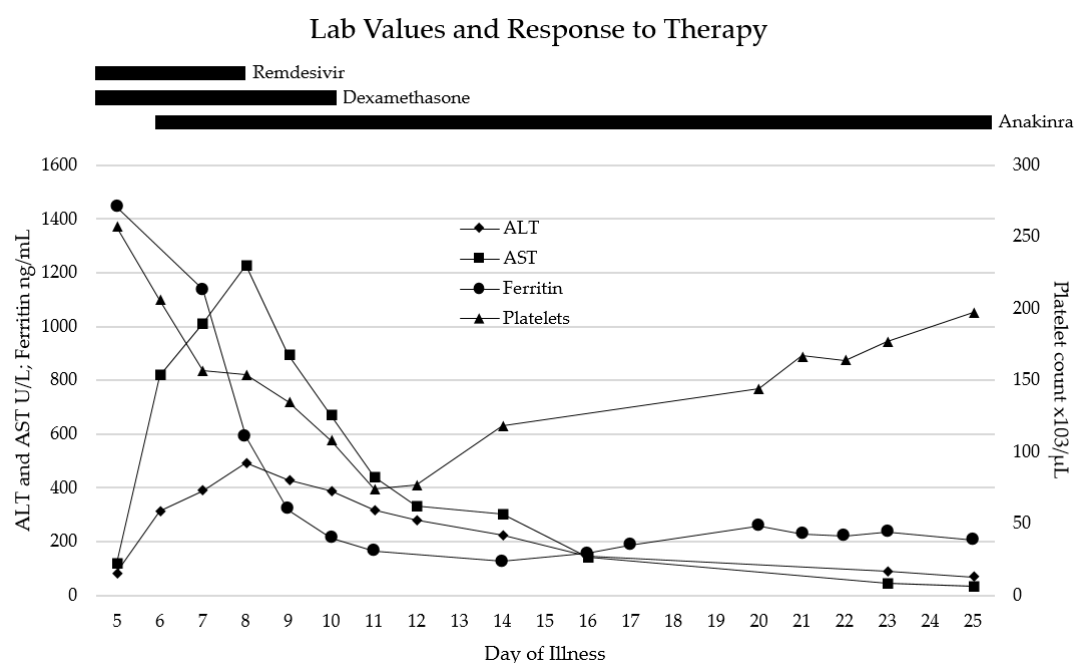
Thrombocytopenia and significant elevation in ALT, AST, and ferritin levels were present on admission, consistent with HLH and end organ damage. Shortly after initiation of corticosteroid and anakinra therapy, laboratory abnormalities rapidly improved throughout her hospital stay.

For the few days after admission (illness days 5–10), the patient remained severely ill, intubated with mechanical ventilation on ECMO and CRRT. She completed 3 days of remdesivir (stopped early due to liver enzyme elevation) and 5 days of dexamethasone treatments for SARS-CoV-2 infection, and received one dose (2 g/kg) of intravenous immunoglobulin (IVIG) due to a low IgG level. By illness day 10, she developed significant rhabdomyolysis with creatinine kinase (CK) max level of up to 67,000 U/L (normal 29–168), maximal aspartate aminotransferase (AST) level of 1226 U/L (normal 13–26), alanine transaminase (ALT) level of 494.2 U/L (normal 8–22), and lactate dehydrogenase (LDH) level of 1836 U/L (normal 130–250). Despite this, the patient’s overall clinical status slowly and steadily improved, and she was able to be slowly weaned off of ECMO by illness day 12 and off all vasopressors by illness day 18. Select laboratory values over time are shown in Figure 1. Anakinra was continued for the majority of hospitalization; the wean was started after discontinuation of vasopressors, and the patient was weaned off of anakinra by illness day 26. She remained admitted after discontinuation of all cardiorespiratory support and immunomodulatory medications due to slow renal recovery and the need for intermittent hemodialysis and rehabilitation services. She was discharged home on illness day 38 with close outpatient follow-up.

Due to the severity of the patient’s presentation, especially the hyperferritinemia and significant cardiac dysfunction, immune markers, and genetics were sent to evaluate for underlying predisposing defects. An Invitae Primary Immunodeficiency Panel resulted with multiple variants of unknown significance, but one of interest was found, a heterozygous STXBP2 (c.1286C > T, p.Ala429Val) mutation associated with autosomal recessive familial hemophagocytic lymphohistiocytosis type 5. No other pHLH gene variants were identified. sHLH markers, soluble IL-2 receptor alpha chain (CD25), and soluble CD163 levels were sent on illness day 22 (after the patient had largely recovered) and were within normal limits—sCD25 622 (normal 137–838 U/mL) and sCD163 785 (normal 387–1785 ng/mL). However, a CD107a degranulation assay was sent from her blood, which showed abnormally low degranulation—CD107a+ NK cells 7% (normal 11–35%) and CD107a mean cell fluorescence (MCF) 121 (normal 207–678)—and an NK cell cytolytic function assay noted as “profoundly decreased to absent NK cell function”, despite being weaned off corticosteroids by that time.

At her one-month follow-up, she continued to be asymptomatic off immunosuppressive medications and was recovering well from a rehabilitation standpoint. Blood counts, liver and kidney function, electrolytes, and inflammatory markers remained normal or trending towards normal. However, a repeat NK cell function assay of her blood continued to show profoundly decreased NK cell function, and a repeat CD107a assay likewise showed stable abnormal findings—CD107a+ NK cells 8% (normal 11–35%) and CD107a MCF 304 (normal 207–678). Further repeat of these assays at the two-month follow-up appointment showed continued decrease in NK cell function, but CXCL9 (a marker of IFNγ) and sCD25 levels were within normal limits.

## 4. Results

### STXBP2 Mutation (p.Ala429Val) Decreases NK Cell Degranulation and Target Cell Lysis

**Figure 2:**
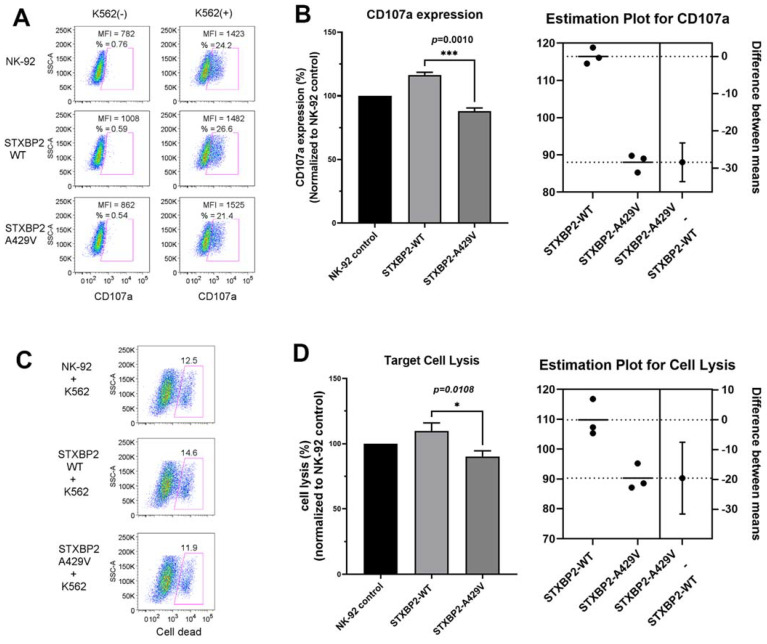
*STXBP2* p.Ala429Val mutation decreases NK cell degranulation and cytolytic function. (**A**) CD107a expression was detected by FCM in NK-92 controls and NK-92 cells transduced with lentiviruses expressing *STXBP2* WT and *STXBP2* p.Ala429Val, with or without co-incubation with K562 cells. Percentages of CD107a+ cells and CD107a mean fluorescence intensities (MFI) are noted. (**B**) CD107a expression was normalized to NK-92 controls, showing a statistically significant difference in expression between *STXBP2* WT and *STXBP2* p.Ala429Val transduced cells. (**C**) eFluor 450-labeled K562 target cells were mixed with NK-92 control cells, NK-92 cells transduced with *STXBP2* WT, and NK-92 cells transduced with *STXBP2* p.Ala429Val. FCM analysis of cell death was performed by near-IR dye staining depicted along the x-axis. (**D**) Cell lysis was normalized to NK-92 control cells, showing a statistically significant difference in cell lysis between *STXBP2* WT and *STXBP2* p.Ala429Val transduced cells. (**A**,**C**) depict one representative example of three independent experiments summarized in (**B**,**D**). * = *p* <0.05; *** = *p* <0.01.

With the notable persistent low NK cell function noted in our patient, her STXBP2 p.Ala429Val mutation was further explored. The STXBP2-encoded protein Munc18-2 is critical for fusion of the trafficked lytic cell cytolytic granule (containing perforin and granzyme B) to the cell membrane via control of soluble NSF (N-ethylmaleimide-sensitive factor) attachment protein receptor (SNARE) complex assembly, and the release of cytotoxic granules by NK cells into the immunologic synapse with the target cell. Using the lentiviral approach, STXBP2 WT (comparative control) and STXBP2 p.Ala429Val mutant cDNA were independently expressed in the human NK cell line NK-92, which already expresses WT STXBP2. Measurement of CD107a cell surface expression is a reliable marker of NK cell degranulation. Degranulation was assessed by FCM as detailed previously in Methods. In the absence of K562 target cells for stimulation, CD107a expression was expectedly minimal on NK-92 cells infected with lentivirus expressing WT or mutant STXBP2 (Figure 2A, left column). Control NK-92 cells (empty lentiviral infection), STXBP2 WT, and STXBP2 p.Ala429Val NK-92 cells were then incubated with K562 target cells, stimulating cytotoxic granule degranulation, leading to increased CD107a expression (Figure 2A, right column). When normalized to NK-92 control CD107a expression, the STXBP2 WT cells had increased expression on the cell surface, but the mutated STXBP2 p.Ala429Val NK-92 cells had notably lower CD107a expression, which was statistically significantly different from WT, as shown in the estimation plot (Figure 2B).

To assess the STXBP2 mutation’s ability to alter target cell death via the perforin pathway, the three cell groups as above were again incubated with K562 target cells. Cell death was assessed using FCM and Live/Dead near-IR dye that enters dead or dying cells, with FCM plots shown in Figure 2C. Percentage of cells lysed was normalized to NK-92 negative controls, with STXBP2 WT transduced NK-92 cells showing increased cell lysis compared to controls, but the STXBP2 p.Ala429Val cells showing significantly decreased lysis compared to WT (Figure 2D).

## 5. Discussion

The hyperinflammation and severe disease associated with secondary HLH has a typical pattern of symptomology and laboratory findings, regardless of its underlying trigger. Persistent fever, multi-lineage cytopenias, liver and kidney end-organ damage, coagulopathy, hypertriglyceridemia, hypofibrinogenemia, elevated CRP with relatively low ESR, and hyperferritinemia can all be seen in cases of HLH [11]. Multiple HLH diagnostic criteria have been used to aid in diagnosis, using a combination of the above clinical characteristics and lab values, with HLH-2004 and HScore as the most commonly used [12,13]. Although there is significant overlap in sHLH and COVID-19 with respect to clinical features and laboratory findings, the frequency of these findings within the overall COVID-19 population and severe COVID-19 subset is still unclear, and there are limited studies dedicated to this line of inquiry. In a collection of 60 severe COVID-19 patients, fever was seen in 71%, hepatosplenomegaly in 24%, cytopenias in 48%, thrombocytopenia in 48%, high ferritin in 96.6% with 75% above 1000 ng/mL and 25% over 10,000 ng/mL, hypertriglyceridemia in 47.2%, and low fibrinogen in 27.2% [14]. Despite these HLH-consistent findings, only 13.3% and 11.6% of patients met the HLH-2004 and HScore criteria, respectively. This relatively limited utility in existing HLH scoring systems for diagnosing sHLH in the setting of COVID-19 has been repeatedly found in the literature, despite the significant overlap in lab values and clinical features [15,16,17]. It may be that the hyperinflammation in severe COVID-19 may be relatively unique, under the cytokine storm syndrome umbrella, but with significant differences from the accepted definitions of sHLH [2]. COVID-19 specific hyperinflammatory and cytokine storm syndrome scoring systems have been developed, which help fill the diagnostic gap left by the more general sHLH diagnostic criteria [18,19,20]. In the case of the patient reported herein, the general and COVID-19 specific cytokine storm scoring systems were inconsistent in their diagnosis (Table 2) with values seen in Table 1.

Even though the patient reported herein inconsistently met criteria for cytokine storm syndrome and sHLH, interleukin-1 directed therapy with anakinra effectively treated her inflammation in the setting of a short course of corticosteroids. Cytokine blockade of IL-1 and IL-6 with anakinra, canakinumab, and tocilizumab are used routinely in treatment of sHLH/MAS [21]; and more recently, cytokine directed therapies have been approved and used effectively in the treatment of severe COVID-19, further increasing evidence of a hyperinflammatory component during high disease activity. Tocilizumab, an IL-6 directed therapy, has been given Food and Drug Administration (FDA) emergency use authorization (EUA) for treatment of hospitalized adults and pediatric patients (2 years of age or older) with significant efficacy and safety shown in randomized controlled trials [22,23]. Anakinra has not received EUA or approval in COVID-19 treatment by the FDA. However, there is evidence of lower risk of clinical progression of COVID-19 with use of anakinra, with further studies ongoing [24].

In addition to responsiveness to sHLH therapies, there are mounting reports of genetic influence on COVID-19 severity, allowing for possible prediction of disease course and early intervention. Differences in haplotypes of killer-cell immune-globulin-like receptors (KIRs) has been shown to be informative of disease course, with the KIR2DS2/HLA C1 function unit protective against COVID-19 adverse outcomes [25]. Conversely, heterozygous mutations, largely missense, in pHLH genes have been correlated with severe COVID-19 symptomology and clinical outcomes [6]. Mutations in *STXBP2* have been previously shown to cause pHLH via homozygous germline mutations [26]. *STXBP2* homozygous mutations in patients diagnosed with pHLH result in lymphoblasts with decreased STXBP2 protein expression and impaired cytotoxic granule exocytosis of NK cells [27,28]. Similarly, monoallelic mutations in *STXBP2* have been shown to contribute to sHLH syndromes via complete or partial dominant-negative effects [4,29]. As seen in the severe COVID-19 patient reported herein, the *STXBP2* p.Ala429Val mutation contributed to decreased NK degranulation and cell lysis ability. In a pediatric MAS cohort of 13 patients, five were found to have *STXBP2* mutations, two of whom had multiple *STXBP2* mutations [4]. Case reports of other *STXBP2* monoallelic mutations have shown similar decreases in NK function and degranulation. A patient with an *STXBP2* (c.568C > T, p.Arg190Cys) mutation developed sHLH in the setting of Langerhans cell histiocytosis; NK cell lysis was found to be decreased, improving with sHLH therapies and CD107a expression was significantly lower than controls [30]. A different study noted that patients with *STXBP2* (c.194G > A, p.Arg65Gln) and *STXBP2* (c.193C > T/p.Arg65Trp) monoallelic mutations again showed decreased degranulation and cytotoxic activity with interference in membrane fusion and SNARE-complex assembly [29]. In addition to these findings, it appears that *STXBP2* mutations in the pediatric population may lead to an increased risk of morbidity and mortality. In a study of anakinra’s effectiveness in treating pediatric sHLH, 5 of 44 patients were noted to have at least 1 monoallelic *STXBP2* mutation and these mutations conferred a statistically higher risk of death in this cohort [31]. Thus, heterozygous missense mutations in perforin pathway genes important for lymphocyte cytolytic activity, including *STXBP2*, may contribute to disease severity of infectious disease such as COVID-19.

Lastly, the patient reported herein had significant cardiac manifestations related to her COVID-19 infection and sHLH, as seen in her elevated cardiac enzyme markers and echocardiography findings. Cardiovascular complications have been frequently reported in adult COVID-19 patients, with myocarditis, myocardial injury and infarction, heart failure, cardiomyopathy, and dysrhythmias seen with varying frequencies [32,33]. SARS-CoV-2 may cause direct damage to heart tissue via angiotensin-converting enzyme 2(ACE2) receptors, but COVID-19 infection in general increases cardiovascular demand, as does increased cytokine activity [33]. It is difficult to tease out cardiac injury caused by direct infectious effect and injury caused by the hyperinflammation seen in sHLH, as sHLH can likewise cause a variety of cardiac manifestations, including myocardial inflammation and cardiogenic shock [34].

## 6. Conclusions

The overlap between severe COVID-19 and sHLH in its clinical features and laboratory findings is significant, as COVID-19 can be an adept infectious trigger of sHLH, capable of creating a severe hyperinflammatory response in genetically predisposed individuals. A subset of severe COVID-19 patients may in fact be harboring these otherwise undiscovered monoallelic pHLH gene mutations. Larger population-based studies are needed to further explore the effect of pHLH genetic mutations on COVID-19 severity and pathogenesis. In doing so, we may find classes of patients who would be good candidates for targeted HLH-based therapies and be able to more effectively treat their disease, limiting morbidity and mortality where possible.

## Figures and Tables

**Table 1:** Select laboratory values on presentation, hospital transfer, and maximum/minimum values.

Laboratory Value	Initial Value Illness Day 3	Hospital Transfer Illness Day 5	Maximum	Minimum
White blood cell count—×10^3^/µL	9.63	23.15	51.09	-
Absolute neutrophil count—×10^3^/µL	8.16	21.53	47.52	-
Absolute lymphocyte count—×10^3^/µL	0.95	1.16	-	0.36
Hemoglobin—g/dL	14.1	13.9	-	7.6
Platelet count—×10^3^/µL	268	257	-	57
C-reactive protein—mg/dL	2.6	2.95	2.95	-
Erythrocyte sedimentation rate (ESR)—mm/h	18	47	47	-
Ferritin—ng/mL	66	1442.9	1442.9	-
Alanine transaminase (ALT)—U/L	10	84.6	494.2	-
Aspartate aminotransferase (AST)—U/L	28	119	1226	-
Creatinine—mg/dL	0.8	1.31	5.38	-
Troponin I—ng/mL	0.696	2.82	91.06	-
Brain natriuretic peptide—pg/mL	-	1498.4	2714.9	-
Creatine kinase-U/L	-	1234	66929	-

**Table 2:** Cytokine storm syndrome scores on admission and at maximal disease activity during hospitalization.

CSS/HLH/MAS System	On Presentation	During Hospitalization
HLH-2004 [10]	No	Yes
H-Score [11]	No-126	No-166
COVID-19 CSS Quick Score [13]	No	Yes
Caricchio COVID-CS Criteria [14]	No	No
COVID-19 cHIS Criteria [15]	5/6	5/6

## Data Availability

Data available upon request to corresponding author.
